# A multimodal intervention to improve hand hygiene compliance in peripheral wards of a tertiary care university centre: a cluster randomised controlled trial

**DOI:** 10.1186/s13756-020-00776-9

**Published:** 2020-07-18

**Authors:** Seven Johannes Sam Aghdassi, Christin Schröder, Elke Lemke, Michael Behnke, Patricia Manuela Fliss, Carolin Plotzki, Janina Wenk, Petra Gastmeier, Tobias Siegfried Kramer

**Affiliations:** 1 Charité – Universitätsmedizin Berlin, corporate member of Freie Universität Berlin, Humboldt-Universität zu Berlin, and Berlin Institute of Health, Institute of Hygiene and Environmental Medicine, Berlin, Germany; 2 National Reference Centre for Surveillance of Nosocomial Infections, Berlin, Germany; 3 BODE SCIENCE CENTER, BODE Chemie GmbH, Hamburg, Germany; 4 Aktion Saubere Hände, Berlin, Germany

**Keywords:** Hand hygiene, Multimodal intervention, Randomised controlled trial, Infection prevention, Non-intensive care ward

## Abstract

**Background:**

Interventions to improve hand hygiene (HH) compliance are a key element in the practice infection prevention and control. It was our objective to assess the effect of a multimodal intervention on HH compliance at a tertiary care university hospital. As a secondary objective, we investigated the effect of the intervention on the occurrence of device-associated bloodstream infections.

**Methods:**

We performed a single centre cluster randomised controlled trial at a university hospital in Germany. Twenty peripheral wards were invited to participate and randomly assigned to either the intervention (*n* = 10) or control group (*n* = 10). Quarterly, specifically trained student employees conducted direct compliance observations in all twenty wards. The intervention entailed dissemination of teaching materials on aseptic procedures, equipment with flexibly mountable alcoholic hand rub dispensers, and quarterly feedback on HH compliance.

**Results:**

In total, 21,424 HH opportunities were observed. Overall, compliance did not change significantly in either group (intervention group: 59% vs. 61% (1482 HH actions for 2494 HH opportunities vs. 5033 HH actions for 8215 HH opportunities), odds ratio (OR) 1.08 (95% confidence interval (CI95) 0.88, 1.33)); control group: 59% vs. 60% (1457 HH actions for 2484 HH opportunities vs. 4948 HH actions for 8231 HH opportunities), OR 1.06 (CI95 0.84, 1.35)). Compliance prior to aseptic procedures improved significantly in the intervention group from 44% (168 HH actions for 380 HH opportunities) to 53% (764 HH actions for 1452 HH opportunities) (OR 1.40 (CI95 1.04, 1.89), *p* = 0.03), while no significant increase was noted in the control group. In the intervention group, significantly fewer device-associated bloodstream infections per 1000 patient-days occurred than in the control group (84 vs. 123, incidence rate ratio 0.61 (CI95 0.46, 0.81), *p* < 0.01).

**Conclusions:**

The lack of a significant overall improvement of HH compliance demonstrated that comprehensive implementation of HH interventions in multiple wards simultaneously is difficult. However, through targeted intervention measures, we were able to significantly increase HH compliance before aseptic procedures.

## Background

Hand hygiene (HH) is one of the most effective measures to prevent healthcare-associated infections (HAIs) and transmission of multidrug-resistant pathogens [1–3]. The implementation of a multimodal strategy as recommended by the WHO, can effectively increase HH compliance and thereby reduce HAIs [4–6]. Implementation of such strategies has been promoted by the German national clean care is safer care campaign (“Aktion Saubere Hände”) for numerous years [7]. Despite these efforts, recent data from the German national HH surveillance network shows a considerable potential for improvement [8]. This especially applies to HH compliance prior to aseptic procedures, which is lower when compared to the other moments [9]. Existing evidence suggests that additional performance feedback and goal setting are effective ways to achieve improved HH compliance [10–15].

We hypothesised that an intensified multimodal intervention (i.e. improving knowledge on HH, dissemination of teaching materials for aseptic procedures, improved accessibility of alcoholic hand rub dispensers at point of care), with the addition of quarterly direct HH observation and feedback would improve HH compliance, and correlate with lower rates of bloodstream infections (BSIs), as well as positive blood cultures overall. We decided to test our hypotheses in a cluster randomised study setting. The stated objectives pertained to the cluster level.

## Methods

### Study design and randomisation

Interventions to promote HH on intensive care units have been conducted at our hospital in the past. On non-intensive care (i.e. peripheral) wards of our hospital, interventions as performed in this trial have not been undertaken previously. We therefore decided to perform a cluster randomised controlled trial in 20 peripheral wards of the Charité-University Medicine hospital in Berlin, a large tertiary care university hospital with three separate sites and approximately 3000 beds. Each participating ward represented a cluster.

In the process of ward selection, we decided to exclude palliative and paediatric wards for ethical reasons. Moreover, intermediate care units were excluded. Since one of our objectives was to assess the effect of the intervention on the incidence of positive blood cultures (see below), we decided to exclude wards with low blood culture sampling frequencies. To achieve that, we evaluated the blood culture sampling frequency of all non-intensive care wards in the year 2016. Wards with a number of blood cultures per 1000 patient-days above the median were included in the randomisation. Of these wards, 20 were randomly selected and allocated to either group (intervention: *n* = 10; control: *n* = 10) with a computer-generated sequence (https://www.randomizer.org/). The randomisation was performed by the study coordinators. Participation of wards was on a voluntary basis. The decision for or against participation was made by the head nurse and physician of the respective ward. Recruitment of wards was performed between September and November 2017. All wards were free to end their participation in the study at any stage without stating a reason. The study was approved by the ethics commission of Charité-University Medicine Berlin (EA4/123/17).

### Description of the intervention

The intervention of this study consisted of three aspects. First, quarterly interdisciplinary team meetings of ward staff with the study team including data on HH compliance observations, and goal setting. Second, distribution of training materials (see description below), and third, distribution of flexibly mountable alcoholic hand rub dispensers. The selection of the individual elements of the intervention was done in alignment with available literature [10–13, 16]. These intervention measures were undertaken and provided solely for wards of the intervention group, not the control group. The intervention period was from the beginning of January 2018 until the end of December 2018.

#### Hand hygiene compliance observations

After randomisation of wards, 5 cycles of HH compliance observations according to the recommendations of the WHO [17], were performed in all 20 wards. The first cycle was performed in December 2017 and served as a baseline assessment against which the effects of the intervention were measured. The following 4 cycles were performed quarterly over the intervention period of 1 year. A minimum of 150 observations with a minimum of 30 observations before clean or aseptic procedure per cycle was required. While staff in control wards received no feedback on HH observation data, wards in the intervention group received regular feedback on compliance data. The feedback was embedded in interdisciplinary team meetings, where the study team presented results of the compliance observations that were then discussed with the ward staff.

HH compliance observations were executed by student employees, which were trained in HH observation methodology by experienced local infection control staff in a specifically organised workshop. Observations were recorded using the Observe app from HARTMANN. In order to increase the quality of observations, the student employees were supervised by an experienced infection control nurse.

#### Intervention materials

Wards randomised into the intervention group were introduced to the intervention during a “kick-off meeting” (i.e. feedback on the first cycle of HH compliance observation), where they received materials to promote HH and infection prevention that had been developed by the study team prior to the study. Wards were encouraged during the kick-off meeting and following quarterly team meetings, as well as by email reminders, to implement these materials into their routine patient care. Among the materials were 10 step-by-step pictogram checklists for selected aseptic procedures, two explanatory films, and flexibly mountable alcoholic hand rub dispensers. The materials were chosen based on the fact that HH is an integral part of many aseptic procedures in routine patient care.

The 10 step-by-step checklists addressed the following topics:

- insertion of peripheral venous catheters (PVCs)
- use of a sterile extension set for PVCs
- management (incl. dressing change) of PVCs
- management (incl. dressing change) of central venous catheters (CVCs)
- management of central venous ports
- preparation of intravenous injections
- application of intravenous injections
- preparation of intravenous infusions
- application of intravenous infusions
- disconnection of intravenous infusions

The two animated explanatory films contained additional information on the insertion of PVCs, and preparation as well as handling of intravenous infusions. All intervention materials were handed out during the kick-off meeting, where applicable, both in printed and electronic form.

### Observed outcomes

As our primary outcome, we selected HH compliance of healthcare workers in participating wards. Compliance was determined by direct observations following the WHO Five Moments of Hand Hygiene model [18].

As a secondary outcome, we selected the incidence of device-associated BSIs for which prospective surveillance was conducted over a 12 months period (11 months during the intervention period, 1 month post-intervention). Due to technical difficulties, only 19 of the 20 participating wards were included in this part of the study. BSIs were defined as a blood culture with a recognised pathogen, or two positive blood cultures with a common skin contaminant, drawn at least 48 h after admission. The two positive blood cultures with a common skin contaminant had to be from two separate blood samples within a five-day period. BSIs were considered as device-associated, if an intravenous catheter was present either on the day of sampling, or the day before in case the catheter was removed on the day of sampling. Furthermore, the catheter had to be in place for a minimum of 3 days before the sample was taken. Where more than one catheter fulfilled the criteria for a device-associated BSI, an association was made to the catheter being inserted in the larger blood vessel (e.g. CVC was chosen over PVC), except where local signs of infection indicated otherwise. Information to make this allocation was gathered from the ward staff, primarily the treating physicians.

Additionally, we evaluated the frequency of positive blood cultures taken at least 48 h after admission of the patient to the ward. For this, all positive blood culture sets of all 20 wards were collected for a one-year baseline period, the one-year intervention period, and a three-month follow-up period. Isolated microorganisms from blood cultures were divided into nine categories: coagulase-negative staphylococci, *Staphylococcus aureus*, *Streptococcus* spp., *Enterococcus* spp., *Enterobacteriales*, non-fermenting bacteria, *Candida albicans*, other *Candida* spp., and others.

All observed outcomes pertained to the cluster level.

### Statistical analysis

HH compliance was calculated for every group (control vs. intervention) and every cycle. HH opportunities and actions were analysed descriptively. To compare the baseline period (cycle 1) with the intervention period (cycles 2–5), the absolute change in compliance was calculated. Additionally, odds ratios (ORs) were calculated with a generalised linear mixed effect model including ward as a random effect. To minimise the potential distortion by confounders and investigate the effect of the intervention on HH compliance more accurately, a multivariable logistic regression analysis was conducted, taking into account other known factors of influence [4, 9, 14, 19]. For this purpose, ORs and 95% confidence intervals (CI95) were calculated. Parameters included in the model were: period (baseline vs. intervention), professional group, WHO-moment, and ward specialty.

Mean infection rates per 1000 patient-days were calculated with CI95 and mid *p*-values. To compare isolates per patient-days between baseline, intervention, and follow-up periods, incidence rates and incidence rate ratios (IRRs) with CI95 and mid *p*-values were calculated. Incidence rates were calculated as Poisson rates. CI95 and *p*-values for IRRs were calculated by median-unbiased estimation. A *p*-value of less than 0.05 was considered significant. All analyses were performed with SAS and R [20]. Graphics were done with ggplot2 [21].

## Results

All 20 wards initially enrolled in the study participated for the full duration. Table S1 in the online supplement (Additional file 1) summarises structural characteristics of the 10 wards in the intervention group and 10 wards in the control group.

**Fig. 1:**
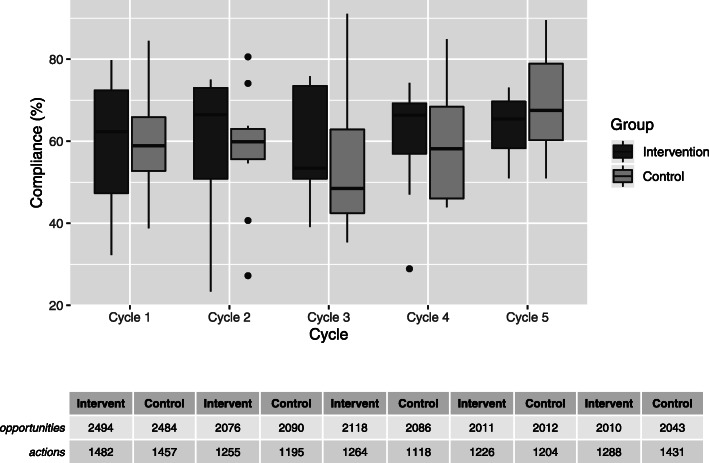
Boxplots of hand hygiene compliance per measurement cycle by study group. The table under the graph lists the number of hand hygiene opportunities and actions per cycle by study group

Overall, 21,424 HH opportunities and 12,920 HH actions were observed. Figure 1 illustrates the compliance per measurement cycle as well as the underlying number of HH opportunities and actions, separately for the intervention and control group. While both groups showed fluctuations in HH compliance over time, median compliance in both groups was higher in cycle 5 than in the baseline measurement (cycle 1). An overall slight increase in HH compliance was also noted when comparing compliance of cycle 1 (baseline period) to aggregated compliance of cycle 2–5 (intervention period). Stratification by the different WHO-moments yielded diverse results. Through further stratifying the indication “before clean or aseptic procedure” by procedure, we were able to detect an increase in compliance before intravascular catheter manipulation (45% vs. 55%) and before contact with mucous membrane (47% vs. 57%) in the intervention group. In the control group, we observed an increase in compliance before preparation of intravenous medication (42% vs. 66%) (Table S2 in the online supplement (Additional file 1)).

While no significant change over time was observed in either group for the overall HH compliance, we observed a significant increase in compliance for the indication “before clean or aseptic procedure” in the intervention group (44% (168 HH actions for 380 HH opportunities) vs. 53% (764 HH actions for 1452 HH opportunities), OR 1.40 (CI95 1.04, 1.89), *p* = 0.03). Although changes were noted with regard to other WHO-moments, these failed to reach statistical significance (Table 1).

**Table 1:** Change in hand hygiene compliance per indication by study group and by study period

WHO-moment	Group	Period	Number of HH-opportunities	Number of HH-actions	Compliance (%)	Absolute change	Odds ratio^**a**^ (CI95), ***p***-value
All	Intervention	Baseline	2494	1482	59.42		reference
		Intervention	8215	5033	61.27	1.85	1.08 (0.88, 1.33), 0.46
	Control	Baseline	2484	1457	58.66		reference
		Intervention	8231	4948	60.11	1.45	1.06 (0.84, 1.35), 0.62
1 – before touching a patient	Intervention	Baseline	698	393	56.3		reference
		Intervention	2346	1408	60.02	3.72	1.16 (0.86, 1.58), 0.33
	Control	Baseline	658	352	53.5		reference
		Intervention	2130	1201	56.38	2.88	1.12 (0.80, 1.58), 0.50
2 – before clean or aseptic procedure	Intervention	Baseline	380	168	44.21		reference
		Intervention	1452	764	52.62	8.41	1.40 (1.04, 1.89), 0.03
	Control	Baseline	426	193	45.31		reference
		Intervention	1738	905	52.07	6.76	1.31 (0.93, 1.84), 0.12
3 – after body fluid exposure risk	Intervention	Baseline	171	114	66.67		reference
		Intervention	527	333	63.19	−3.48	0.86 (0.60, 1.23), 0.40
	Control	Baseline	241	145	60.17		reference
		Intervention	705	482	68.37	8.20	1.43 (0.94, 2.18), 0.09
4 – after touching a patient	Intervention	Baseline	791	563	71.18		reference
		Intervention	2496	1774	71.07	−0.11	1.00 (0.77, 1.29), 0.97
	Control	Baseline	668	499	74.7		reference
		Intervention	2414	1685	69.8	−4.90	0.78 (0.56, 1.09), 0.15
5 – after touching patient surroundings	Intervention	Baseline	454	244	53.74		reference
		Intervention	1394	754	54.09	0.35	1.01 (0.81, 1.27), 0.90
	Control	Baseline	491	268	54.58		reference
		Intervention	1244	675	54.26	−0.32	0.99 (0.76, 1.28), 0.92

Abbreviations: *HH* hand hygiene, *CI95* 95% confidence interval; ^a^ result of logistic regression with ward as a cluster effect

To identify factors that had a significant influence on HH compliance, we performed a multivariable logistic regression analysis for the outcome compliant performance of HH. To determine the effect of the intervention, compliance in the intervention period was compared separately for the two study groups to compliance of the baseline measurement. While the intervention period was not revealed to have a significant effect in either of the two study groups, the profession of the healthcare worker and the WHO-moment both had a significant effect on the outcome. The professional group “others” (i.e. non-nursing, non-physician staff) were associated with significantly lower HH compliance. The WHO-moment “after touching a patient” was associated with significantly higher compliance when compared to other WHO-moments (Table 2).

**Table 2:** Multivariable logistic regression model for the outcome compliant hand hygiene

Parameter	Characteristic	Odds ratio^**a**^ (CI95)	***p***-value
Period	Baseline (intervention group)	reference	
	Baseline (control group)	0.92 (0.53, 1.59)	0.75
	Intervention (intervention group)	1.09 (0.87, 1.36)	0.44
	Intervention (control group)	0.97 (0.53, 1.78)	0.93
Professional group	Physicians	reference	
	Others	0.51 (0.32, 0.81)	< 0.01
	Nurses	0.99 (0.79, 1.24)	0.93
WHO-moment	4 – after touching a patient	reference	
	1 – before touching a patient	0.55 (0.46, 0.67)	< 0.01
	2 – before clean or aseptic procedure	0.41 (0.30, 0.57)	< 0.01
	3 – after body fluid exposure risk	0.77 (0.60, 0.99)	0.04
	5 – after touching patient surroundings	0.53 (0.44, 0.63)	< 0.01
Unit specialty	Medical	reference	
	Surgical	1.16 (0.68, 1.99)	0.58

Abbreviations: *CI95* 95% confidence interval; ^a^ result of logistic regression with ward as a cluster effect

Comparison of device-associated BSIs showed that significantly fewer infections per 1000 patient-days occurred in the intervention group versus the control group (84 vs. 123, IRR 0.61 (CI95 0.46, 0.81), *p* < 0.01). When stratifying infections by device, no significant differences were found for PVCs or central venous ports. However, significantly fewer CVC-associated BSIs per 1000 patient-days occurred in the intervention group than control group (36 vs. 75, IRR 0.43 (CI95 0.29, 0.64), *p* < 0.01) (Table 3).

**Table 3:** Device-associated bloodstream infections per 1000 patient-days by study group

Device	Intervention group		Control group		
	Number of BSIs	Mean rate/1000 patient-days (CI95)	Number of BSIs	Mean rate/1000 patient-days (CI95)	IRR (CI95), ***p***-value
All	84	0.71 (0.57, 0.88)	123	1.16 (0.96, 1.38)	0.61 (0.46, 0.81), < 0.01
Peripheral venous catheter	10	0.08 (0.04, 0.16)	11	0.10 (0.05, 0.19)	0.82 (0.34, 1.96), 0.65
Central venous port	20	0.17 (0.10, 0.26)	21	0.20 (0.12, 0.30)	0.86 (0.46, 1.59), 0.62
Central venous catheter	36	0.31 (0.21, 0.42)	75	0.71 (0.56, 0.89)	0.43 (0.29, 0.64), < 0.01
Other	18	0.15 (0.09, 0.24)	16	0.15 (0.09, 0.24)	1.01 (0.51, 2.01), 0.97

Abbreviations: *BSIs* bloodstream infections, *IRR* incidence rate ratio, *CI95* 95% confidence interval

A separate analysis of positive blood cultures taken at least 48 h after admission showed a significantly higher occurrence of positive isolates per 1000 patient-days in the control group during the intervention period versus the baseline period (297 vs. 226, IRR 1.28 (CI95 1.08, 1.53), *p* < 0.01). We did not observe a similar significant change in the intervention group with regard to the study period (Table 4). Data for positive blood cultures separated by pathogen or pathogen-group are illustrated in Table S3 in the online supplement (Additional file 1).

**Table 4:** Positive blood cultures per 1000 patient-days by study group and by study period

Study group	Baseline period		Intervention period		Follow-up period		Comparison		
	Number of isolates	Mean rate/ 1000 patient-days (CI95)	Number of isolates	Mean rate/ 1000 patient-days (CI95)	Number of isolates	Mean rate/ 1000 patient-days (CI95)	IRR (CI95), ***p***-value Intervention vs. Baseline	IRR (CI95), ***p***-value Follow-up vs. Baseline	IRR (CI95), ***p***-value Follow-up vs. Intervention
**Intervention group**	161	1.42 (1.21, 1.66)	172	1.46 (1.25, 1.70)	53	1.78 (1.33, 2.32)	1.03 (0.83, 1.27), 0.80	1.25 (0.91, 1.69), 0.17	1.22 (0.88, 1.64), 0.22
**Control group**	226	1.98 (1.73, 2.26)	297	2.54 (2.26, 2.85)	59	2.08 (1.58, 2.68)	1.28 (1.08, 1.53), < 0.01	1.05 (0.78, 1.39), 0.74	0.82 (0.61, 1.07), 0.15

Abbreviations: *IRR* incidence rate ratio, *CI95* 95% confidence interval

## Discussion

Overall, compliance of healthcare workers to the WHO Five Moments of Hand Hygiene improved in both arms of our study. This increase was not statistically significant and lower than in reports from other randomised controlled trials [14, 22, 23]. A similar study by Stewardson et al., observed a larger overall increase in HH compliance in the different groups of their trial, even with a higher initial baseline compliance than in our study [13]. More in alignment with our results, Fuller et al. observed improved HH compliance in intensive care units, but not in peripheral units [10]. Despite our centre’s long lasting participation in the national clean care is safer care campaign and successful history of implementation of multimodal strategies for infection prevention, direct observations of compliance had not been performed in most of the participating wards prior to this study. However, some wards independently continued the practice of quarterly direct compliance observation and feedback after the end of the trial. We consider this an important achievement underscoring the relevance of a sense of ownership among healthcare workers in any intervention designed to increase patient safety in a sustainable manner.

Despite only a marginal increase in overall HH compliance, we observed a significant improvement of compliance before aseptic procedures in the intervention group, while compliance for this moment improved in the control group as well, but not significantly. This is especially relevant, since the teaching materials provided as part of our intervention strategy primarily focused on this moment. A decision, which was made in reaction to the known low compliance before aseptic procedures [24]. Previous randomised controlled trials have not placed a similar focus on HH compliance before aseptic procedures. The fact that there was no long-term follow-up to our intervention, however, renders it difficult to estimate the sustainability of the observed positive effects. Whether these will persist and whether the improvement in HH compliance, and particularly in HH compliance before aseptic procedures, will be long-lasting, remains to a certain extent speculative.

Multivariable logistic regression revealed that compliance was highly dependent on the type of moment and the profession of the healthcare worker. Compliance “after touching a patient” was higher than for any other moment. These findings correspond to finding of other international and national publications [5, 6, 8]. In alignment with other publicised studies, non-nursing, non-physician staff (“others”) showed significantly lower compliance [5, 8]. Underlying reasons for this phenomenon might be the heterogeneity of this professional group and worse accessibility of training and education on the matter. Multivariable analysis did not identify the period “intervention” as a factor with a significant effect on HH compliance. This missing effect could either be explained by inadequacy of the intervention measures, or insufficient uptake of the intervention by ward staff. Unstructured qualitative feedback from wards and observations made by the study team during the quarterly feedback meetings rather suggested a perceived lack of ownership by ward staff. This may have resulted in an insufficient uptake and implementation of intervention measures by the ward staff (e.g. infrequent use of teaching materials), illustrating the difficulty of establishing an effective intervention in multiple wards simultaneously. The importance of a sense of ownership and enhanced leadership regarding HH have been demonstrated in earlier publications [25, 26].

We observed significantly lower rates of device-associated BSIs in the intervention group when compared to the control group, similar to other studies that have demonstrated a reduction in HAI rates due to improved HH [27–29]. Despite a focus on PVCs in our teaching materials, this difference was mainly due to a lower rate of CVC-associated BSIs. This observation may be attributable to differences in patient population and frequency of CVC-usage. However, since we did not record device-days in the included wards during the study period, this explanation remains speculative. Overall, low rates of PVC-associated BSIs were recorded, reiterating findings from a previous study on the matter at our centre [30].

As an additional finding, we observed that pathogens identified from blood cultures, regardless of whether device-associated or not, significantly increased in the control group during the intervention period when compared to the baseline period. A similar trend was not observed in the intervention group, which can be interpreted as a positive effect of the intervention. Regarding the pathogens obtained from positive blood cultures, no significant changes were observed concerning the most frequently occurring pathogens. Explanations for this result, however, remain speculative, given the overall low number of positive blood cultures per pathogen or pathogen-group.

Various limitations have to be acknowledged when interpreting the data. The direct observations of HH performed in this study only represent a fraction of all HH opportunities and actions performed in wards during the study period. Baseline compliance was established in a single measurement and was therefore prone to potential random effects. Observed shifts in compliance could have set in before the start of the intervention (i.e. between baseline measurement and “kick-off meeting”). Furthermore, the study design did not include a long-term follow-up regarding HH compliance. Therefore, it remains speculative whether observed effects of the intervention were sustainable or not. With regard to potential confounders, it has to be recognised that “cross contamination”, for instance distribution of intervention materials from intervention to control wards was possible, and that the observation itself may have already contributed to improved HH compliance (“Hawthorne effect”) in all wards (incl. Control wards). To minimise these potential confounders, observers did not give feedback to healthcare workers during the observations, and all personnel in the intervention wards were asked to not further distribute any of the provided materials for the duration of the study. Another potential confounder was due to the fact that the primary observers were a heterogeneous group of students who had limited pre-existing knowledge about HH and compliance observation. To address this and increase the robustness of our data, all primary observers were trained in accordance with the WHO HH observation methodology, supervised by an experienced infection control nurse, and steadily assigned to certain wards.

We have no standardised information on how intervention materials were used in the day-to-day routine work of the intervention wards, making it difficult to estimate their effect. Furthermore, rotations and changes in ward staffing, as well as changes in equipment used by wards (e.g. different types of catheters used over time) could represent a confounding factor.

For outcome parameters relating to BSIs, definitions used for device-associated BSIs were not evaluated against established surveillance definitions, since no clinical data on patients, such as fever or other indicators of infection, were systematically collected. Therefore, it is possible that we overestimated BSI-rates. We tried to account for this by including the opinion of the treating physicians in our surveillance. Furthermore, no baseline measurement before the onset of the intervention was available for device-associated BSIs. Consequently, regarding this parameter, solely comparisons between the groups, but not over time, were possible.

## Conclusions

In conclusion, a statistically significant increase in HH compliance prior to aseptic procedures was observed only for wards in the intervention group. Rates of device-associated BSIs as a surrogate for clinically relevant and preventable infections were significantly lower in the intervention group. These findings illustrate an effect of our intervention. However, the lack of a significant overall increase in HH compliance underlines the difficulty of our attempt to establish an effective multimodal intervention in multiple wards simultaneously. Future studies will be required to focus on the barriers of implementation and novel approaches to increase HH compliance, especially outside the intensive care setting.

## Supplementary information

**Additional file 1.**

## Data Availability

The datasets used and/or analysed during the current study are available from the corresponding author on reasonable request.

## Abbreviations

HH: Hand hygiene
HAI: Healthcare-associated infection
BSI: Bloodstream infection
PVC: Peripheral venous catheter
CVC: Central venous catheter
OR: Odds ratio
CI95`: 95% confidence interval
IRR: Incidence rate ratio
