# Supplementary material for: A multimodal intervention to improve hand hygiene compliance in peripheral wards of a tertiary care university centre: a cluster randomised controlled trial
Source: Antimicrob Resist Infect Control. 2020 Jul 18;9:113. doi: 10.1186/s13756-020-00776-9 (PMC7368705; doi:10.1186/s13756-020-00776-9)
Supplement: Supplementary file 1 — Additional file 1. [file 13756_2020_776_MOESM1_ESM.pdf]

A multimodal intervention to improve hand hygiene compliance in peripheral wards of a tertiary care university centre: a cluster randomised controlled trial

Supplementary material

Table S1 Structural characteristics of wards by study group

**Parameter**		**Intervention group**	**Control group**	**Total**
Number of wards (total)		10	10	20
Number of medical wards		9	7	16
	Hematology/Oncology	2	3	5
	Cardiology	1	1	2
	Gastroenterology/ Nephrology	1	3	4
	Neurology	2	0	2
	Mixed/Other	3	0	3
Number of surgical wards		1	2	3
	Abdominal surgery	1	0	1
	Traumatology	0	1	1
	Gynecology/Urology	0	1	1
Number of interdisciplinary wards		0	1	1
Patient-days in the year 2018	Median (Interquartile range)	10545 (8814-14771)	10283 (8425-15143)	10928 (8814-15143)

Table S2 Hand hygiene (HH) opportunities and actions per WHO-moment, profession and aseptic procedure by study group and by study period

	Baseline period (cycle 1)		Intervention period (cycle 2-5)	
	Intervention group	Control group	Intervention group	Control group
WHO-moment	**Number of HH-opportunities; Number of HH-actions; HH-compliance**			
**All**	2494; 1482; 59%	2484; 1457; 59%	8215; 5033; 61%	8231; 4948; 60%
**1 – before touching a patient**	698; 393; 56%	658; 352; 53%	2346; 1408; 60%	2130; 1201; 56%
**2 – before clean or aseptic procedure**	380; 168; 44%	426; 193; 45%	1452; 764; 53%	1738; 905; 52%
**3 – after body fluid exposure risk**	171; 114; 67%	241; 145; 60%	527; 333; 63%	705; 482; 68%
**4 – after touching a patient**	791; 563; 71%	668; 499; 75%	2496; 1774; 71%	2414; 1685; 70%
**5 – after touching patient surroundings**	454; 244; 54%	491; 268; 55%	1394; 754; 54%	1244; 675; 54%
Profession	**Number of HH-opportunities; Number of HH-actions; HH-compliance**			
**All**	2494; 1482; 59%	2484; 1457; 59%	8215; 5033; 61%	8231; 4948; 60%
**Nurses**	1385; 838; 61%	1683; 992; 59%	5269; 3281; 62%	5459; 3374; 62%
**Physicians**	767; 523; 68%	641; 393; 61%	1887; 1232; 65%	2234; 1327; 59%
**Others**	342; 121; 35%	160; 72; 45%	1059; 520; 49%	538; 247; 46%
Aseptic procedure	**Number of HH-opportunities; Number of HH-actions; HH-compliance**			
**All**	380; 168; 44%	426; 193; 45%	1452; 764; 53%	1738; 905; 52%
**Preparation of intravenous medication**	51; 29; 57%	65; 27; 42%	274; 161; 59%	285; 189; 66%
**Intravascular catheter manipulation**	130; 58; 45%	196; 100; 51%	496; 271; 55%	691; 380; 55%
**Insertion of intravascular catheter**	78; 31; 40%	83; 31; 37%	405; 173; 43%	383; 164; 43%
**(Wound-) Dressing change/ Manipulation of drainage**	41; 22; 54%	62; 26; 42%	112; 65; 58%	213; 86; 40%
**Contact with mucous membrane**	30; 14; 47%	18; 8; 44%	139; 79; 57%	148; 75; 51%
**Not specified**	50; 14; 28%	2; 1; 50%	26; 15; 58%	18; 11; 61%

Table S3 Positive blood cultures stratified by pathogen per 1,000 patient-days by study group and by study period

Study group	Pathogen/pathogen-group	Baseline period		Intervention period		Follow-up period		Comparison		
		Number of isolates	Mean rate/ 1000 patient-days (CI95)	Number of isolates	Mean rate/ 1000 patient-days (CI95)	Number of isolates	Mean rate/ 1000 patient-days (CI95)	IRR (CI95), p-value Intervention vs. Baseline	IRR (CI95), p-value Follow-up vs. Baseline	IRR (CI95), p-value Follow-up vs. Intervention
Intervention	All	161	1.42 (1.21, 1.66)	172	1.46 (1.25, 1.70)	53	1.79 (1.34, 2.34)	1.03 (0.83, 1.27), 0.80	1.26 (0.92, 1.71), 0.15	1.23 (0.89, 1.66), 0.20
	Coagulase-negative staphylococci	24	0.21 (0.14, 0.32)	32	0.27 (0.19, 0.38)	10	0.34 (0.16, 0.62)	1.28 (0.76, 2.20), 0.36	1.61 (0.73, 3.28), 0.23	1.25 (0.58, 2.47), 0.54
	Staphylococcus aureus	31	0.27 (0.19, 0.39)	30	0.26 (0.17, 0.36)	4	0.14 (0.04, 0.35)	0.93 (0.56, 1.54), 0.78	0.51 (0.15, 1.29), 0.17	0.55 (0.16, 1.39), 0.23
	Streptococcus spp.	10	0.09 (0.04, 0.16)	14	0.12 (0.07, 0.20)	2	0.07 (0.01, 0.24)	1.34 (0.59, 3.14), 0.48	0.81 (0.11, 3.13), 0.79	0.60 (0.09, 2.19), 0.49
	Enterococcus spp.	18	0.16 (0.09, 0.25)	21	0.18 (0.11, 0.27)	4	0.14 (0.04, 0.35)	1.12 (0.59, 2.13), 0.72	0.88 (0.25, 2.37), 0.81	0.78 (0.22, 2.07), 0.64
	Enterobacteriales	50	0.44 (0.33, 0.58)	45	0.38 (0.28, 0.51)	15	0.51 (0.28, 0.84)	0.87 (0.58, 1.30), 0.48	1.15 (0.62, 2.01), 0.64	1.33 (0.72, 2.34), 0.35
	Non-fermenting bacteria	5	0.04 (0.01, 0.10)	3	0.03 (0.01, 0.07)	5	0.17 (0.05, 0.39)	0.59 (0.11, 2.50), 0.47	3.82 (1.03, 14.20), 0.05	6.49 (1.53, 33.63), 0.01
	Candida albicans	3	0.03 (0.01, 0.08)	7	0.06 (0.02, 0.12)	1	0.03 (0.00, 0.19)	2.18 (0.59, 10.69), 0.25	1.39 (0.05, 11.95), 0.80	0.64 (0.03, 3.67), 0.67
	Other *Candida* spp.	3	0.03 (0.01, 0.08)	3	0.03 (0.01, 0.07)	0	0.00 (0.00, 0.12)	0.96 (0.17, 5.60), 0.96	0 (0, 6.55), 0.50	0 (0, 6.81), 0.51
	Others	17	0.15 (0.09, 0.24)	17	0.14 (0.08, 0.23)	12	0.41 (0.21, 0.71)	0.96 (0.49, 1.91), 0.91	2.71 (1.25, 5.66), 0.01	2.81 (1.30, 5.88), <0.01
Control	All	226	1.98 (1.73, 2.26)	297	2.54 (2.26, 2.85)	59	2.08 (1.58, 2.68)	1.28 (1.08, 1.53), <0.01	1.05 (0.78, 1.39), 0.74	0.82 (0.61, 1.07), 0.15
	Coagulase-negative staphylococci	36	0.32 (0.22, 0.44)	53	0.45 (0.34, 0.59)	9	0.32 (0.14, 0.60)	1.44 (0.94, 2.21), 0.09	1.02 (0.46, 2.03), 0.96	0.71 (0.32, 1.37), 0.32
	Staphylococcus aureus	21	0.18 (0.11, 0.28)	29	0.25 (0.17, 0.36)	3	0.11 (0.02, 0.31)	1.35 (0.77, 2.40), 0.30	0.60 (0.14, 1.75), 0.38	0.45 (0.10, 1.26), 0.14
	Streptococcus spp.	14	0.12 (0.07, 0.21)	20	0.17 (0.10, 0.26)	3	0.11 (0.02, 0.31)	1.39 (0.70, 2.83), 0.34	0.90 (0.20, 2.78), 0.86	0.64 (0.15, 1.89), 0.46
	Enterococcus spp.	31	0.27 (0.18, 0.39)	42	0.36 (0.26, 0.49)	7	0.25 (0.10, 0.51)	1.32 (0.83, 2.12), 0.24	0.92 (0.37, 1.98), 0.85	0.70 (0.28, 1.46), 0.36
	Enterobacteriales	66	0.58 (0.45, 0.74)	85	0.73 (0.58, 0.90)	15	0.53 (0.30, 0.87)	1.26 (0.91, 1.74), 0.16	0.92 (0.50, 1.57), 0.77	0.73 (0.41, 1.23), 0.25

Non-fermenting bacteria	15	0.13 (0.07, 0.22)	15	0.13 (0.07, 0.21)	1	0.04 (0.00, 0.20)	0.98 (0.47, 2.03), 0.95	0.30 (0.01, 1.50), 0.17	0.31 (0.01, 1.53), 0.18
Candida albicans	8	0.07 (0.03, 0.14)	7	0.06 (0.02, 0.12)	4	0.14 (0.04, 0.36)	0.86 (0.30, 2.43), 0.77	2.05 (0.53, 6.64), 0.28	2.39 (0.60, 8.11), 0.20
Other *Candida* spp.	11	0.10 (0.05, 0.17)	12	0.10 (0.05, 0.18)	3	0.11 (0.02, 0.31)	1.06 (0.46, 2.47), 0.88	1.14 (0.25, 3.70), 0.85	1.07 (0.23, 3.41), 0.92
Others	24	0.21 (0.13, 0.31)	34	0.29 (0.20, 0.41)	14	0.49 (0.27, 0.83)	1.38 (0.82, 2.36), 0.22	2.35 (1.18, 4.51), 0.02	1.70 (0.88, 3.12), 0.11

Abbreviations: IRR – incidence rate ratio; CI95 – 95% confidence interval
